# Neuromuscular Junction Disorders and Floppy Infant Syndrome: A Comprehensive Review

**DOI:** 10.7759/cureus.6922

**Published:** 2020-02-08

**Authors:** Jasndeep Kaler, Azhar Hussain, Sundip Patel, Shankar Majhi

**Affiliations:** 1 Medicine, Xavier University School of Medicine, Oranjestad, ABW; 2 Healthcare Administration, Franklin University, Columbus, USA; 3 Medicine, Windsor University School of Medicine, Cayon, KNA; 4 Biochemistry, Xavier University School of Medicine, Oranjestad, ABW

**Keywords:** floppy infant syndrome, floppy baby syndrome, infantile botulism, hypotonia, myasthenia gravis, neonatal toxicity, hyperkalemia

## Abstract

Floppy infant syndrome, also sometimes referred to as rag-doll syndrome, is characterized by hypotonia that could present as either peripheral hypotonia or central. Depending on the origin of hypotonia, the infant will present with different symptoms that ultimately have the characteristic feature of hypotonia. The clinical examination is crucial in diagnosing floppy infant syndrome in the neonate period, but the most critical factor is investigating and diagnosing the underlying cause of hypotonia. Regardless of whether the underlying cause of hypotonia is peripheral or central in origin, the presentation of floppy infant syndrome focuses on observing for the presence or absence of specific signs such as ‘frog-leg’ posture, significant head lag on traction or pull-to-sit maneuver, or the feeling of ‘slipping through the hands’ when the infant is held under the arms. Infantile botulism, transient neonatal myasthenia gravis, congenital myasthenia gravis, hypermagnesemia, and aminoglycoside toxicity are all neuromuscular junction disorders that are considered to be a differential diagnosis of floppy infant syndrome. These neuromuscular junction disorders ultimately impact the presence of acetylcholine within the neuromuscular junction. While some of these disorders may impact the acetylcholine receptors, others may cause a depletion within the end-plate anticholinesterase enzyme. A deficiency within the anticholinesterase deficiency may cause desensitization to acetylcholine, which could also cause present with floppy infant syndrome as well. Depending on the underlying causative disorder leading to the presence of floppy infant syndrome, the treatment will vary considerably. Treatment of the underlying causative syndrome resulting in the presentation of floppy infant syndrome deals with the symptoms of hypotonia, and as a result, the decreased muscle tone, diminished tendon reflexes, any feeding or respiratory difficulties diminish.

## Introduction and background

Floppiness/hypotonia is defined as reduced resistance to passive movement of joints, and clinically, floppy/hypotonic infants exhibit hypotonia along with motor developmental delay, hyperextensibility of joints, abnormal postures [1]. Floppy infant syndrome (FIS) is defined as a decrease in muscular tone that varies in severity and duration. The list of causative factors, ultimately leading to the prevalence of FIS, is long and extensive. The hypotonia present in a floppy infant can be categorized as being central in origin or peripheral [2]. It should be noted that, ultimately, the central nervous system (CNS) disorders are the much more common cause of hypotonia [1]. Conducting a very detailed clinical examination is crucial for physicians to be able to differentiate and diagnose a central or peripheral cause of hypotonia as appropriate differentiation between allows physicians to understand better the underlying cause that is resulting in floppy infant syndrome. Central causes of hypotonia are often associated with a depressed level of consciousness, predominantly axial weakness, normal strength with hypotonia, and hyperactive or normal reflexes, fisting of the hands, scissoring on vertical suspension, and abnormalities of brain function or dysmorphic features [3]. The severity and prevalence of these features in central hypotonia are highly dependent on the underlying causative agent. Some syndromes may present with a broader spectrum of symptoms that remain persistent over the years while most cases of floppy infant syndrome present with decreased muscular tone/hypotonia that tends to cause developmental delays in crucial milestones, however, disappears as the child approaches adolescence. Dysfunction at any level of the nervous system could cause hypotonia, including disorders of the cerebellum, spinal cord, anterior horn cells, peripheral nerves, neuromuscular junctions, and muscles; dysfunction at any of these levels predominantly leads to the development of peripheral hypotonia [1]. If a hypotonic infant is alert, responds appropriately to surroundings, and shows normal sleep-wake patterns, the hypotonia is likely due to involvement of the peripheral nervous system and the peripheral causes are associated with profound weakness in addition to hypotonia, hyporeflexia or areflexia and sometimes feeding difficulties [3]. As with central hypotonia, the true severity and presentation of the symptoms are primarily dependent on the type of underlying cause of the floppy infant syndrome. Whether it be peripheral hypotonia or central hypotonia leading to the presence of floppy infant syndrome, the clinical examination focuses on the presence or absence of specific signs, such as the presence of ‘frog-leg’ posture, significant head lag on traction or pull-to-sit maneuver, rag-doll posture on ventral suspension and the feeling of ‘slipping through the hands’ when the infant is held under the arms [4]. 

While conducting the clinical examination, clinicians must be attentive to situations in which central and peripheral hypotonia symptoms may be comorbid. In conditions where there is a comorbidity of central and peripheral hypotonia symptoms, the severity of the presenting symptoms will be, once again, dependent on the underlying cause of the hypotonia. Some of these conditions where peripheral and central hypotonia may co-exist are hypoxic-ischemic encephalopathy, lipid storage diseases, lysosomal disorders, mitochondrial disorders, and infantile neuroaxonal degeneration [5].

Table 1 presents a visual representation of the differences in the symptoms that are present in peripheral hypotonia versus central hypotonia. Although there are varying symptoms amongst the two, ultimately, both can lead to the presentation of floppy infant syndrome, and the duration of this syndrome varies depending on the underlying cause. Causes of central hypotonia may have some symptoms that remain throughout life, such as the myopathic facies or the relatively mild social or cognitive impairment that might be present. Other factors, such as the frog-leg posture or reduced tendon jerks, may dissipate over time. The dissipation of these types of symptoms may not occur until after the motor milestones have been reached, which usually are delayed in comparison to the normal infants. Because of the presence of delayed motor milestones, central hypotonia is more prevalent around 1-2 years of age as the parents notice that their child is not walking or crawling and due to such, may present to the clinician’s office with the chief complaint of delayed motor milestones. The same can be applied in peripheral hypotonia as some symptoms may be more stagnant to the individual’s lifestyle while other symptoms may decrease in intensity and slowly disappear altogether as time progresses. Dysmorphic features or other organ malformations may remain as a static feature of peripheral hypotonia, whereas seizures may decrease in intensity into adolescence and sometimes disappear altogether. As mentioned earlier, it is imperative to diagnose the underlying cause of floppy infant syndrome to provide the appropriate care and therapy, and sometimes treatment to ensure that the child’s lifestyle is maintained, and supportive care is provided in a way that allows FIS patients to maintain or increase their muscular tone. Nutrition is also of primary importance to maintain ideal body weight for the age and sex through the nasogastric route as patients with hypotonia can present with feeding difficulties as well [2,5]. Through this paper, we will be focusing on neuromuscular junction disorders that lead to peripheral hypotonia and, thus, the presentation of floppy infant syndrome. We will be providing a brief overview of the condition, the pathophysiology, and pathogenesis of the condition, along with the presentation of hypotonia and any possible treatments or therapy. The objective of this paper is to provide an appropriate amount of information on the neuromuscular junction disorders and their varying presentations of hypotonia that lead to the secondary diagnosis of FIS.

**Table 1:** Different Presentations between Peripheral and Central Hypotonia

Indicators of Peripheral Hypotonia	Indicators of Central Hypotonia
Social and cognitive impairment, in addition to motor delay; Dysmorphic features implying a syndrome or other organ malformations sometimes implying a syndrome; Fisting of hands; Normal or brisk tendon reflexes; Crossed adductor response or scissoring present upon vertical suspension; Features suggestive of an underlying spinal dysraphism; Seizures; History that is suggestive of hypoxic-ischemic encephalopathy, birth trauma or symptomatic hypoglycemia	Delay in motor milestones with relative normality of social and cognitive impairment; Family history of neuromuscular disorders/maternal myotonia; Reduced or absent spontaneous antigravity movements reduced or absent deep tendon jerks and increased range of joint mobility; Frog-leg posture or ‘jug-handle’ posture of arms in association with marked paucity of spontaneous movement; Myopathic facies (open mouth with tented upper lip, poor lip seal when sucking, lack of facial expression, ptosis and restricted ocular movements)

## Review

About 50% of the cases of hypotonia are successfully diagnosed with just a proper history and physical examination, including obtaining a family history, maternal obstetric history, clinical and neurological examinations [3]. When looking at the causes of peripheral hypotonia, a diagnostic workup is based on an understanding of the anatomy of the motor unit as many causes of peripheral hypotonia can be localized to various compartments of the motor unit [2].

**Figure 1:**
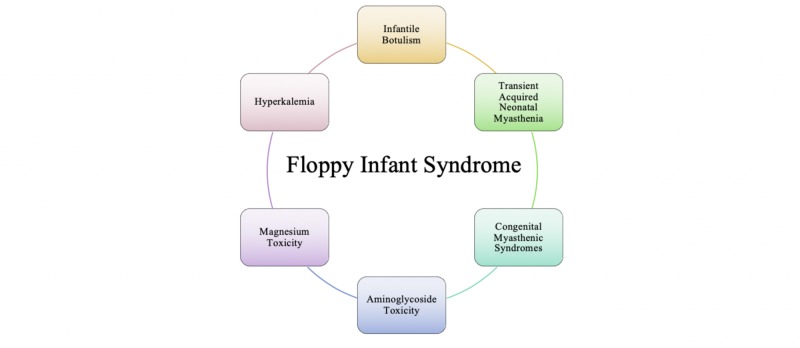
Neuromuscular Junction Disorders Causing Floppy Infant Syndrome

Figure 1 is a visual depiction of the neuromuscular junction disorders that we will be focusing on as causes of floppy infant syndrome. Neuromuscular junction disorders are denoted as causes of peripheral hypotonia that share several features, including hypotonia, facial diplegia, ptosis, feeding difficulties, apnea, respiratory difficulties, generalized weakness, and progressively weakening cry [6]. Each of these neuromuscular junction disorders will be discussed to establish the pathogenesis of hypotonia and the presentation that leads to floppy infant syndrome.

Infantile botulism 

Infantile botulism, caused by consumption of contaminated honey or corn syrup in 20% of the cases, is an age-limited disorder in which Clostridium botulinum (C. botulinum) is ingested, colonizes the intestinal tract, and produces the toxin in situ [3]. Infantile botulism usually occurs within six weeks to one year after birth, and the first symptom these infants present with is often constipation [6]. According to Cagan et al. (2010), the United States Centers for Disease Control and Prevention (CDC) reviewed the reported cases of botulism in all of its forms in the United States between 1899 and 1996 and found that 1442 cases of infant botulism were reported in 46 states between 1976 to 1996 [7]. C. botulinum is a gram-positive, spore-forming, obligate anaerobe that is present in the soil worldwide and may spread by dust [8]. The symptoms associated with infantile botulism are all due to the enteric toxins released by C. botulinum. The botulinum toxin is the most potent neurotoxin that does not appear to cross the blood-brain barrier; however, it exerts its toxicity by affecting the transmission at all peripheral cholinergic junctions by interfering with the normal release of acetylcholine from nerve terminals in response to depolarization [9]. The enteric toxin causes intestinal immobility and progressive descending paralysis due to the effect on acetylcholine release at the neuromuscular junction and other cholinergic nerve terminals, particularly in the gut [7,10]. Infantile botulism differs from food-borne botulism in the sense that with food-borne botulism, there is ingestion of a preformed toxin in contrast to infantile botulism in which there is continued intra-intestinal production of toxin due to clostridial colonization of the large intestine. Historically, infants afflicted with botulism are between 2 and 26 weeks of age, usually live in a dusty environment adjacent to construction or agricultural soil disruption [3]. As mentioned earlier, while the first symptom of infantile botulism is constipation, other symptoms such as listlessness, ptosis, facial weakness, decreased eye movements, feeding difficulties, and progression to respiratory failure can occur [6].

**Figure 2:**
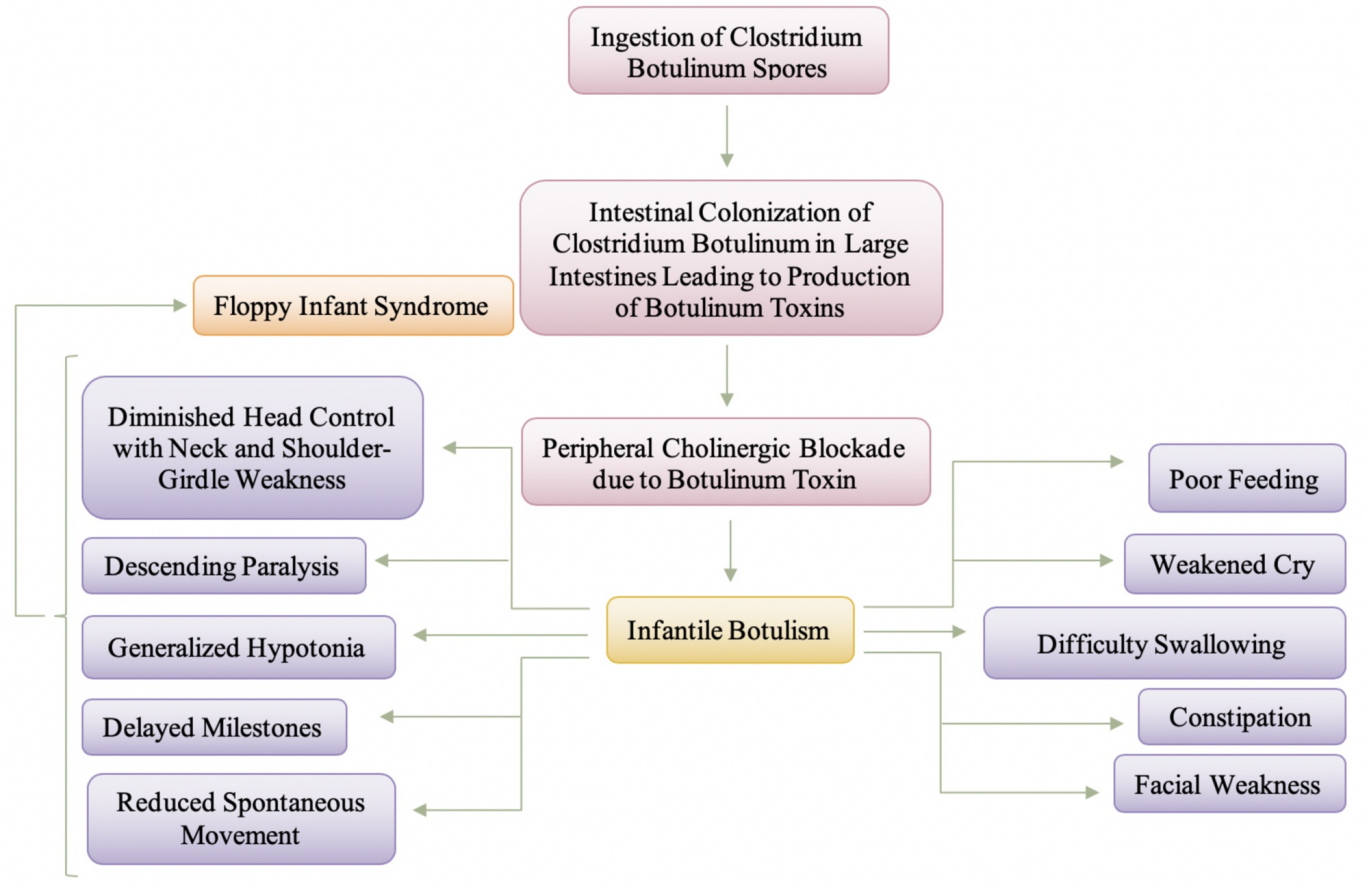
Pathogenesis and Manifestation of Infantile Botulism

Figure 2 provides a visual representation of the pathogenesis and manifestation of infantile botulism. As depicted in Figure 2, the colonization of C. botulinum causes the continuous production of the botulinum toxin within the intestinal tract, specifically the large intestines of the infant as a byproduct of the germination and multiplication of the spores of Clostridium botulinum. The produced botulinum toxin is then absorbed and undergoes hematogenous distribution through which the toxin binds presynaptically at the neuromuscular junction and other peripheral cholinergic synapses, thereby preventing acetylcholine release [11]. Colonization and germination of the spores of C. botulinum in the infant’s gut are possible due to the lack of normal gut flora. The infantile intestinal tract lacks protective bacterial flora and Clostridium-inhibiting bile acids, which allows the C. botulinum to flourish and produce the toxin that causes the disease [10]. The prevention of acetylcholine release is what leads to the clinical effect, which is hypotonia and descending, symmetric flaccid paralysis [11]. The generalized hypotonia and other clinical manifestations are owing to progressive neuromuscular blockade, initially of muscles innervated by cranial nerves and later of the trunk, extremities, and diaphragm [8]. The generalized hypotonia can also precipitate a delay in motor milestones and a reduction in spontaneous movements due to the absence of reflexes. Alongside the generalized hypotonia, expressionless face, decreased gag reflex, difficulty swallowing, and poor suck are also primary features of infantile botulism [3].

The clinical manifestations of infantile botulism can be plotted on a spectrum, depending on the severity of the disease. The key clinical manifestation of infantile botulism, as mentioned earlier, is constipation which is defined as three or more days without a bowel movement, followed by a subacute progression of bulbar and extremity weakness that manifests as an inability to suck and swallow, weakened voice, ptosis, hypotonia that progresses to generalized flaccidity and eventually, respiratory compromise [8]. The generalized weakness presents as a descending paralysis that occurs over hours to a few days and begins with weakness in the innervation of the cranial nerves to those of trunks and limbs [6,8]. Progression of infantile botulism is more severe in infants younger than two months, suggesting a more severe form of hypotonia being present [9]. The diagnosis of infantile botulism is made on clinical grounds and is confirmed by identification of the neurotoxin in the stool, when necessary by sterile enema [8]. Furthermore, an enzyme-linked immunosorbent assay (ELISA) has recently been developed for rapid detection of toxins in infantile botulism that allows for detection to be possible within 24 hours as compared to four days that are required for the mouse assay [8,9]. Children presenting with infantile botulism presenting with mild symptoms require minimal care and can be managed as outpatients if careful follow-up is arranged [8]. Infants with severe infantile botulism constitute a select group who are at risk for respiratory failure, and these infants can be identified by their progressive sequential loss of neurological functions [8,9]. Seriously ill patients require hospitalization for up to two months, and during this period, careful maintenance of adequate ventilation and caloric intake is important, and the need for respiratory assistance, if any, generally occurs during the first week of hospitalization [8].

Antibiotics are not recommended for infantile botulism and do not affect the course of the illness or the recovery of the disease; however, there is some argument that effective antibiotics may increase the pool of toxin in the bowel available for absorption as it is liberated following bacterial cell death [8,11]. Brook (2007) mentions that another argument against the use of antimicrobial agents is that these agents may alter the intestinal microecology in an unpredictable manner and might permit intestinal overgrowth by C. botulinum by eliminating the normal flora [8]. The present treatment of infantile botulism consists of meticulous supportive care, with particular attention to nutrition, pulmonary hygiene, and good nursing care [5]. The prognosis of infantile botulism is generally excellent, and because of this, the association of the disease with floppy infant syndrome is treatable. As the infantile botulism symptoms are reversed, the presence of FIS will dissipate as well.

Congenital myasthenia

Congenital myasthenic syndromes result from gene mutations affecting the neuromuscular junction structure and function [12]. Infants presenting with the myasthenia syndrome share several features, including hypotonia, facial diplegia, ptosis, feeding difficulties, apnea, respiratory difficulties, generalized weakness, and a progressively weakening cry, making congenital myasthenia syndrome a differential diagnosis of floppy infant syndrome [6]. Congenital myasthenic syndromes can present at any time from birth to adulthood, though usually within the first two years of life, and result in a spectrum of diseases ranging from mild weakness to severe disability with life-threatening episodes [13]. Congenital myasthenia is an umbrella term for a category of syndromes that all impact the neuromuscular junction but differ in whether the deficiency is due to presynaptic, synaptic, or postsynaptic defects of neuromuscular transmission which leads to either an increased response to acetylcholine or a decreased response. Despite the causative agent of the syndrome, ultimately, the problem arises from the lack of acetylcholine or the inability of properly working acetylcholine receptors. For our purposes, and in order to make the association with floppy infant syndrome, we will be focusing our attention onto the end-plate acetylcholinesterase deficiency, the most common form of synaptic congenital myasthenic syndrome.

With end-plate acetylcholinesterase deficiency, a study by Engel et al. (1977) described a patient whose symptoms began soon after birth and included generalized weakness, increased by exertion, easy fatiguability, hyporeflexia, and refractoriness to anticholinesterase drugs [14]. The lack of appropriate acetylcholine available causes the generalized weakness and hyporeflexia to present as floppy infant syndrome and, therefore, causes congenital myasthenia to be presented as a differential diagnosis of FIS. The first symptoms usually arise in the neonatal period, and the symptoms are severe with a significant lethal risk; however, the disease may start later, during infancy, and is not so severe [15]. End-plate acetylcholinesterase (AChE) is an enzyme that is responsible for the rapid hydrolysis of acetylcholine (ACh) released at cholinergic synapses [16]. Deficiency of end-plate acetylcholinesterase causes acetylcholine to linger in the synapse for a longer than normal period of time, causing the acetylcholine receptors to become desensitized to the acetylcholine present. The desensitization leads to a higher amount of acetylcholine to be released in order for the same response to be initiated. Acetylcholinesterase deficiency is related to mutations in the COLQ gene coding for the collagenic tail of acetylcholinesterase [15]. End-plate acetylcholinesterase (AChE) consists of globular catalytic subunits attached to the basal lamina by a collagen-like tail, and different genes encode the catalytic subunit and the tail portion of the enzyme [16]. The collagenic tail concentrates and anchors the enzyme within the synaptic basal lamina [15].

**Figure 3:**
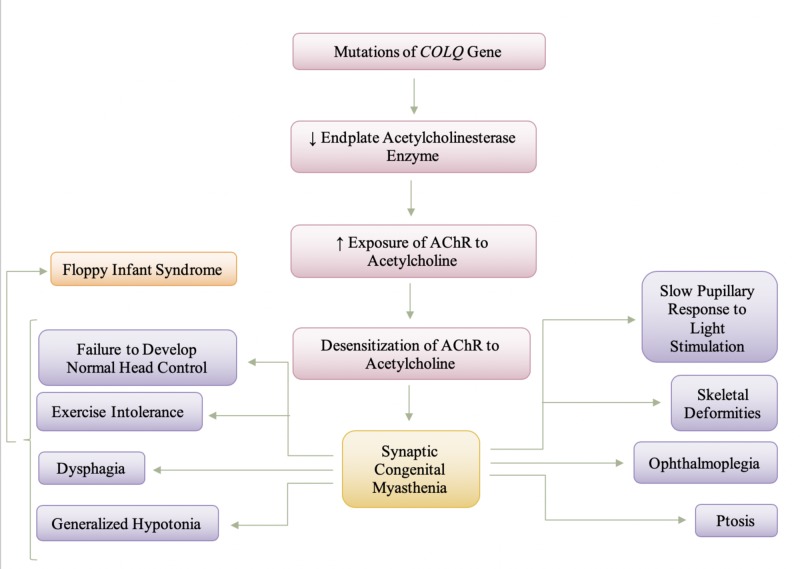
Pathogenesis and Manifestation of Synaptic Congenital Myasthenia

Figure 3 depicts the pathogenesis and manifestation of synaptic congenital myasthenia. It should be noted, however, that symptoms and presentation of synaptic congenital myasthenia vary from individual to individual, depending on the severity of the deficiency of acetylcholinesterase. Individuals presenting with a slight deficiency of the acetylcholinesterase enzyme may present with a slightly less severe phenotype in comparison to those that may present with a greater deficiency. The generalized hypotonia also varies in presentation amongst individuals. A study conducted by Hutchinson et al., (1993) reports a case of a 4-month-old infant that presented with respiratory insufficiency immediately after birth and failed to develop normal head control, presented with hyporeflexia in arms and diffuse weakness [16]. Infants with synaptic congenital myasthenia syndrome present with increased weakness in muscles that is visible following exercise, thus, causing exercise intolerance. The presentation of floppy infant syndrome secondary to the decreased end-plate acetylcholinesterase enzyme is noticeable as not only weakness in the limb muscles but could also present as truncal/axial hypotonia. The dysphagia, feeding difficulties, maybe a secondary symptom that an infant could present with due to truncal/axial hypotonia. Skeletal deformities (ex. lordosis or scoliosis), ptosis, ophthalmoplegia, dysphagia, limb weakness, and difficulty breathing can occur with disease progression [17]. To date, there is no effective pharmacologic treatment available for this subtype of congenital myasthenic syndrome; however, some patients have demonstrated partial improvement with the use of ephedrine or albuterol [16,17]. The exact mode of action of ephedrine or albuterol is not fully understood in humans, although it has been hypothesized that they increase the amount of acetylcholine released and reduce the acetylcholine receptor open time in-vitro [17]. Cholinesterase inhibitors are contraindicated and should be avoided in synaptic congenital myasthenia because they may increase respiratory secretions and contribute to possible end-plate myopathy, a toxic effect of calcium [15,17].

Transient acquired neonatal myasthenia

Transient acquired neonatal myasthenia occurs in infants born to mothers with myasthenia gravis in which the acetylcholine receptor antibody that causes myasthenia gravis crosses the placenta and exerts a blocking effect that is responsible for the interference with neuromuscular transmission [6]. Neonatal transient myasthenia gravis is a self-limited disorder that may be potentially life-threatening if prompt and accurate diagnosis and supportive respiratory management are not initiated [18]. There is a natural passive transfer of maternal antibodies that cross the placenta and bind to fetal motor-end plates, specifically against the nicotinic acetylcholine receptor (AChR) [19,20]. Transient neonatal myasthenia was reported in 12.26% of infants born to mothers with generalized myasthenia gravis before the discovery and use of AChR antibody titers for diagnosis of acquired autoimmune myasthenia gravis [18]. The anti-AChR antibodies that are passed onto the fetus through the placenta will passively induce the loss of AChRs, leading to impaired neuromuscular transmission causing muscle weakness as the primary symptom [21]. The reduced number of AChRs causes a decreased sensitivity to acetylcholine at the end-plate. Due to this mechanism, the end-plate potentials (EPPs) can be so low that threshold for activating the voltage-gated sodium channels is not reached, and consequently, no action potential is generated [19,21].

**Figure 4:**
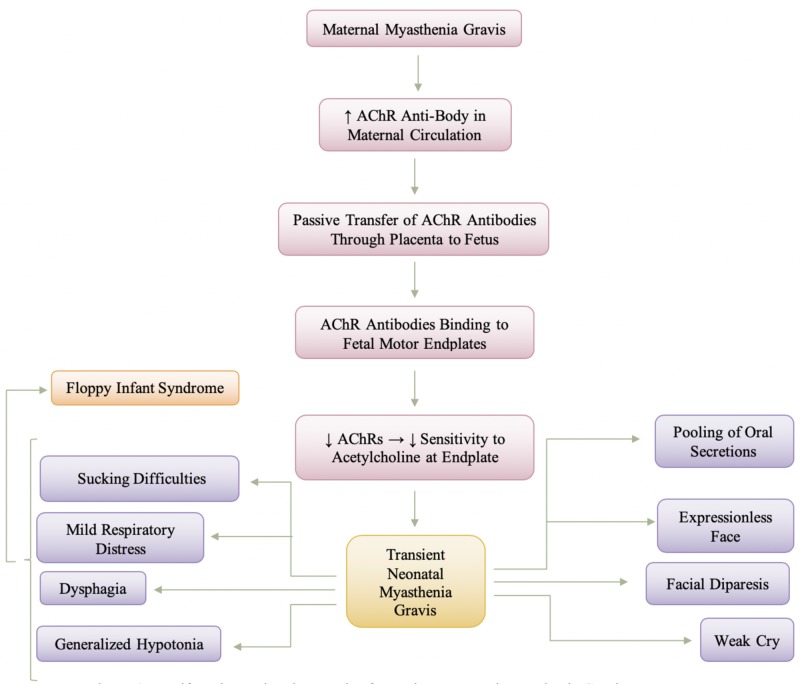
Manifestation and Pathogenesis of Transient Neonatal Myasthenia Gravis

Other symptoms besides muscle weakness, shown in Figure 4, include weak sucking, dysphagia, feeble cry, hypotonia, and, more rarely, respiratory difficulty, and are evident within the first two days of life and generally lasts 2-4 weeks [19]. There also may be a slight delay after birth before the symptoms may appear [2]. Constant clinical findings in the common form are poor sucking and generalized hypotonia, and other manifestations are weak cries, facial diparesis with an expressionless face, swallowing and sucking difficulties, and mild respiratory distress [18]. Infants are typically severely hypotonic, causing neonatal transient myasthenia gravis to become a differential diagnosis for floppy infant syndrome; however, this hypotonia is typically not long-term. The generalized weakness is usually associated with facial diplegia and the pooling of oral secretions [2]. Treatment is symptomatic; assisted ventilation, exchange transfusion, and intravenous immunoglobulins (IVIg) are rarely needed [19]. For the most part, the transmitted disease is short-lived (days to usually weeks), which reflects the biologic decay of circulating antibody and regeneration of normal binding protein at the myoneural junction of the baby, usually disappearing after six weeks [2,22]. In the meantime, the infant will benefit from symptomatic care and anticholinesterase drugs [2]. In infants where the symptoms are moderate to severe, neostigmine methylsulfate can be administered intramuscularly, subcutaneously or through a nasogastric tube 20 minutes and 30 minutes before feeding, respectively and it should be noted that the dose may be increased gradually until sucking and swallowing are adequate to meet the infant’s nutritional needs [18]. It should also be noted that once the infant is asymptomatic, the use of anticholinesterase drugs should be slowly decreased and, ultimately, stopped.

Aminoglycoside toxicity

Aminoglycosides are a mainstay of antimicrobial therapy for infants in cases in which infections are due to gram-negative bacteria, accounting for up to 25% of all sepsis episodes in neonatal units [23]. Aminoglycosides have a narrow therapeutic window, and close monitoring is required to minimize potential nephrotoxicity, ototoxicity, neuromuscular blockade [24]. The antibacterial effect of aminoglycosides is afforded mainly by their binding to the 30S ribosomal subunit, leading to the misreading of RNA, disruption of protein synthesis, and, ultimately, accumulation of truncated and nonfunctional proteins leading to bacterial death [25,26]. The RNA interactions are relatively selective for bacterial ribosomes because structural differences lower the drugs’ affinity for eukaryotic ribosomes and allow for generally safe human use [26]. Aminoglycosides cause an antibiotic-induced neuromuscular blockage that can result in a decrease in the postjunctional acetylcholine sensitivity or a decrease in the release of acetylcholine in the presence of neomycin or gentamicin, specifically [27]. Even within the category of aminoglycosides, gentamicin presents with the highest potency in terms of neuromuscular blocking activity [28]. Out of the aminoglycosides, gentamicin, neomycin, streptomycin, tobramycin, and kanamycin have been reported to produce clinically significant muscle weakness on occasion in non-myasthenia gravis patients [27]. Most evidence indicates that aminoglycosides possess both pre- and postjunctional blocking actions, but the reversal of the blockade by either calcium or anticholinesterase agents is unpredictable [29]. Aminoglycoside toxicity is a concern of greater magnitude in premature infants and neonates, and this is mainly due to the renal system being immature and thus, resulting in a prolonged serum half-life of the aminoglycosides. The prolonged serum half-life will manifest as nephrotoxicity, ototoxicity, and also cause neuromuscular blockade resulting in muscle weakness, generalized hypotonia - ultimately making aminoglycoside toxicity a differential diagnosis of floppy infant syndrome.

Aminoglycosides are also contraindicated in myasthenia-like syndromes and infantile botulism, as these drugs tend to worsen the conditions. Myasthenia-like syndromes and infantile botulism are all neuromuscular junction disorders, as explained above in the paper, and because aminoglycosides have the potential to cause a neuromuscular blockage as well, such drugs could worsen the hypotonia present in these conditions. Amongst the various drugs, different types of aminoglycosides impact the neuromuscular junction at different points; while some may be impacting the presynaptic release of acetylcholine, others may decrease the acetylcholine receptors present on the postsynaptic membrane. Many in vitro and in vivo animal studies have shown that aminoglycoside antibiotics potentiate the action of nondepolarizing muscle relaxants and these findings have been correlated in humans by many case reports where aminoglycoside antibiotics either increased the duration of action muscle relaxants or caused recurrence of neuromuscular blockade produced by d-tubocurarine, pancuronium or vecuronium after reversal of the block [30]. The flaccid paralysis prevalent from the neuromuscular blockade from aminoglycosides is rare, although the greatest risk is associated with rapid intravenous administration of the aminoglycosides. Aminoglycoside antibiotics, along with calcium channel blockers, interfere with calcium ion movements through the calcium channels of the membranes of the motor nerve-endings inhibiting acetylcholine release at the synaptic cleft and thus, could lead to respiratory depression, prolonged apnea and/or flaccid paralysis [31].

**Figure 5:**
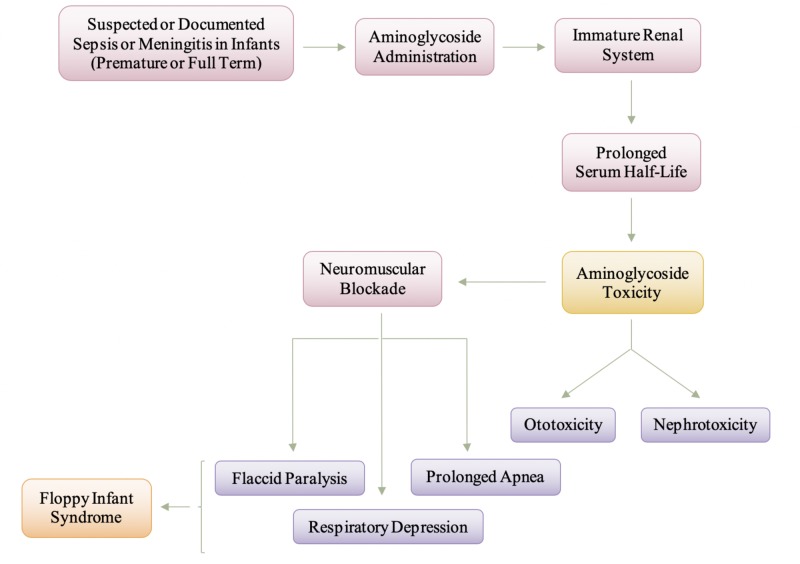
Manifestation and Pathogenesis of Aminoglycoside Toxicity in Infants and Neonates

Figure 5 is a depiction of the manifestation and pathogenesis of aminoglycoside toxicity. Aminoglycosides are typically administered in infants where there is a suspected or confirmed case of sepsis and meningitis, and this is of greater concern in premature infants. The pathology of nephrotoxicity primarily involves the proximal tubules, a major site of drug accumulation, but can be clinically managed with hydration therapy so that patients generally recover normal renal function once treatment with aminoglycosides is discontinued [32]. Ototoxicity induced by aminoglycosides, on the other hand, manifests as irreversible bilateral sensorineural hearing loss beginning at high frequencies (cochleotoxicity), or as any combination of vertigo, nausea, vomiting, nystagmus, and ataxia (vestibulotoxicity) [26]. The effect of aminoglycosides at the neuromuscular junction is still being researched as the two greater concerns of aminoglycoside toxicity are ototoxicity and nephrotoxicity. As mentioned earlier, different aminoglycosides tend to impact the neuromuscular junction at different levels - presynaptic or postsynaptic. The impact at the presynaptic terminal is associated with a decrease in acetylcholine being released, and the impact at the postsynaptic terminal is associated with a decreased sensitivity at the acetylcholine receptors. Ultimately, the aminoglycosides will cause a neuromuscular blockade causing a decrease in acetylcholine and decreased sensitivity to the acetylcholine present. The decrease in acetylcholine in the neuromuscular junction will cause myasthenia-like symptoms, specifically causing flaccid paralysis and fatal respiratory depression. The flaccid paralysis and respiratory depression are the symptoms associated with aminoglycoside toxicity that bring it to be a differential diagnosis of floppy infant syndrome. The impact of the aminoglycosides at the neuromuscular junction is completely reversed with the administration of calcium chloride [31]. In animal models, neuromuscular blockade was shown to be rapidly reserved by the administration of calcium gluconate, and in some cases, supportive care alone will resolve the blockade [31].

Magnesium toxicity

Elevated magnesium levels can be encountered in the newborn following the treatment of maternal eclampsia with magnesium sulfate or following the use of magnesium antacids in the newborn, resulting in an encephalopathic infant with hypotonia, depressed deep tendon reflexes, abdominal distension due to ileus and irregularities of cardiac rhythm [6]. Hypermagnesemia is defined as a serum magnesium concentration greater than 1.15 mmol/L (2.8 mg/dL) [33]. Women with pre-eclampsia are at risk of developing seizures, which are associated with adverse outcomes for the mother and the fetus, and therefore, anti-convulsant treatments such as magnesium sulfate are given to mothers with eclampsia to reduce the risk of seizures and improve outcome [34]. Neonatal hypermagnesemia can be caused by increased magnesium load such as maternal magnesium sulfate administration, newborn magnesium therapy, or decreased renal magnesium excretion due to prematurity or asphyxia [35]. After administration, about 40% of plasma magnesium is protein-bound, and the unbound magnesium ion diffuses into the extravascular-extracellular space, into bone, and across the placenta and fetal membranes and into the fetus and amniotic fluid [36]. Due to magnesium ions being present within the amniotic fluid, not only is the infant taking in the magnesium through fetal membranes but is also actively ingesting the magnesium through the ingestion of the amniotic fluid. Infants born to mothers with pre-eclampsia or eclampsia who received magnesium sulfate can have hypermagnesemia presenting with generalized hypotonia, apnea, bradycardia, feeding difficulty, and, in severe cases, respiratory distress and may even mimic septic shock [33]. The combination of symptoms that the infants present with could be described as floppy infant syndrome. The feeding difficulty and respiratory distress could be allocated to the generalized hypotonia causing decreased muscle tone and, thus, decreased muscular movements. Magnesium is known to inactivate acetylcholine at the neuromuscular junction, especially in the respiratory muscles, and does not affect the brain directly [2,33]. At the neuromuscular junction, magnesium sulfate decreases the amount of acetylcholine liberated, diminishes the sensitivity of the end-plate to acetylcholine, and depresses the excitability of the muscle membrane thus, resulting in skeletal muscle weakness and respiratory distress [37]. Calcium entry into the presynaptic terminal is necessary for acetylcholine release, and magnesium competitively blocks calcium entry [38].

**Figure 6:**
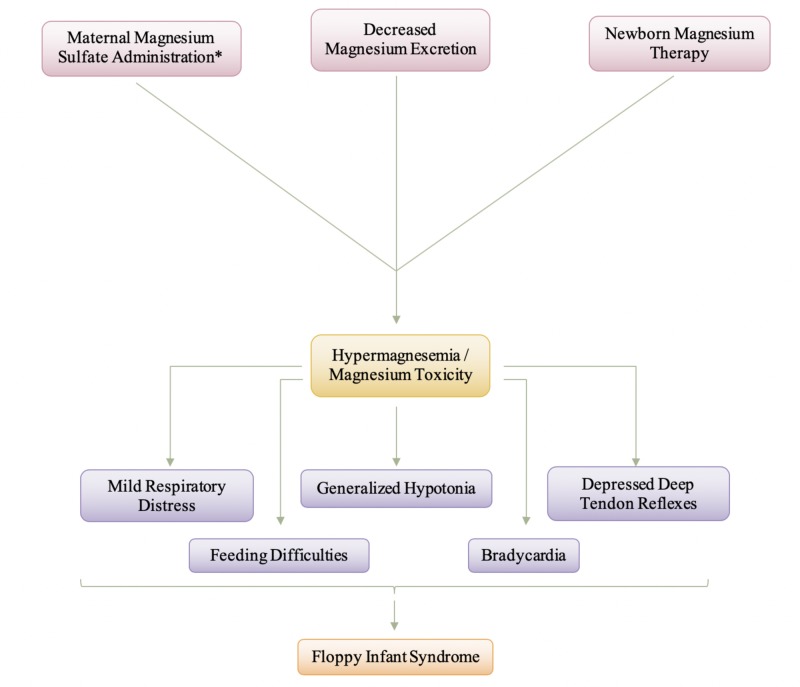
Manifestation and Pathogenesis of Neonate Magnesium Toxicity

Figure 6 is a depiction of the pathogenesis and manifestation and neonate magnesium toxicity. As shown in Figure 6 by the asterisk, maternal magnesium sulfate administration is the most common cause of magnesium toxicity in a neonate. A combination of symptoms, specifically characterized by generalized hypotonia, causes hypermagnesemia to be a differential diagnosis of floppy infant syndrome. Lipsitz and English (1967) reported respiratory depression and hyporeflexia in a small group of newborn infants of mothers treated with intravenous MgSO4 [39]. It should be noted that the severity of hypotonia that is prevalent within the affected infant will be very dependent on the level of serum magnesium that crosses the placenta barrier and is delivered to the infant. For most infants, treatment of hypermagnesemia is close monitoring and supportive care, while the body eliminates the excess magnesium by urinary excretion [33]. Intravenous fluids may also be given to optimize hydration and increase urinary flow rate, or loop diuretics may be given to aid in the renal elimination of excess magnesium [33,37]. In acute cases, intravenous calcium may also be given as a direct inhibitor of magnesium [33]. With the appropriate treatment and once the excess magnesium has been eliminated, the symptoms and the presentation of floppy infant syndrome will dissipate. The generalized hypotonia, hyporeflexia, and feeding difficulties will begin to become less frequent and eventually be gone.

Hyperkalemia

Hyperkalemia is present in up to 52% of premature infants with a birth weight of less than 1000g, and hyperkalemic infants are at a high risk of developing life-threatening cardiac arrhythmias [40]. Non-oliguric hyperkalemia is characterized ours after by an excessive increase in serum potassium concentration at 24 hours after birth and is mainly due to the immature functioning of the sodium (Na^+^)/potassium (K^+^) pump [41]. The early-onset hyperkalemia may have been caused by the accumulation of potassium ions transported through the placenta, the shift of potassium ions from the intracellular to the extracellular space in the infant due to the malfunctioning of the Na^+^/K^+^ pump and the inhibition of renal distal tube potassium ion secretion [42]. Non-oliguric hyperkalemia of the premature infant is a result of a loss of potassium into the extracellular space to the extent that only occurs during the first days after birth in very immature infants, and it has been suggested that this potassium loss is secondary to an immature function of the Na^+^/K^+^ pump [40]. Another explanation to the cause of the early onset of hyperkalemia is maternal hyperkalemia caused by hypermagnesemia since, usually, the fetal plasma K^+^ ion concentration is higher than the maternal plasma concentrations [42]. The causes of hyperkalemia in infants are numerous, and therefore, to pinpoint one specific cause of hyperkalemia deems to be challenging. The causes of hyperkalemia in infancy include acute hemolysis, kidney disorders, and hormonal disorders [43]. For this paper, our focus is on the cause of hyperkalemia in the neuromuscular junction, and for this purpose, we will be focusing on how maternal hypermagnesemia can also cause hyperkalemia in an infant.

Potassium is the second most abundant cation in the body, with about 98% of potassium being intracellular and 2% of the body’s potassium in the extracellular fluid, where the concentration is tightly regulated [44]. Magnesium is a modulator of the ion transport systems in numerous tissues, and hypermagnesemia inhibits K^+^ ion transport from the extracellular to the intracellular space through the Na^+^/K^+^ pump [42]. Additionally, hypermagnesemia inhibits renal distal tube K^+^ ion secretion by the renal outer medullary K^+^ (ROMK) channel, which is an inward-rectifying K^+^ responsible for basal K^+^ ion secretion [42,45]. In the case of maternal and fetal hypermagnesemia, the underlying mechanism of this hyperkalemia is mainly assumed to be secondary to hypermagnesemia and subsequent malfunctioning of the Na^+^/K^+^-ATPase, and inhibition of secretion in the renal outer medullary K^+^ (ROMK) channel [42]. The presence of hyperkalemia causes neuromuscular junction disorders in these infants, such that during the action potential, the repolarization phase is prolonged. Because of a prolonged phase of repolarization, the next action potential takes much longer to occur. Due to a longer refractory period and the inability of the next action potential to generate at the same magnitude, the infant would present with muscle weakness and, thus, hypotonia. Ultimately, it should be noted that hyperkalemia does not directly lead to the presentation of floppy infant syndrome, but because it causes muscle weakness and paralysis, it can be associated with causing hypotonia, which is largely associated with floppy infant syndrome.

**Figure 7:**
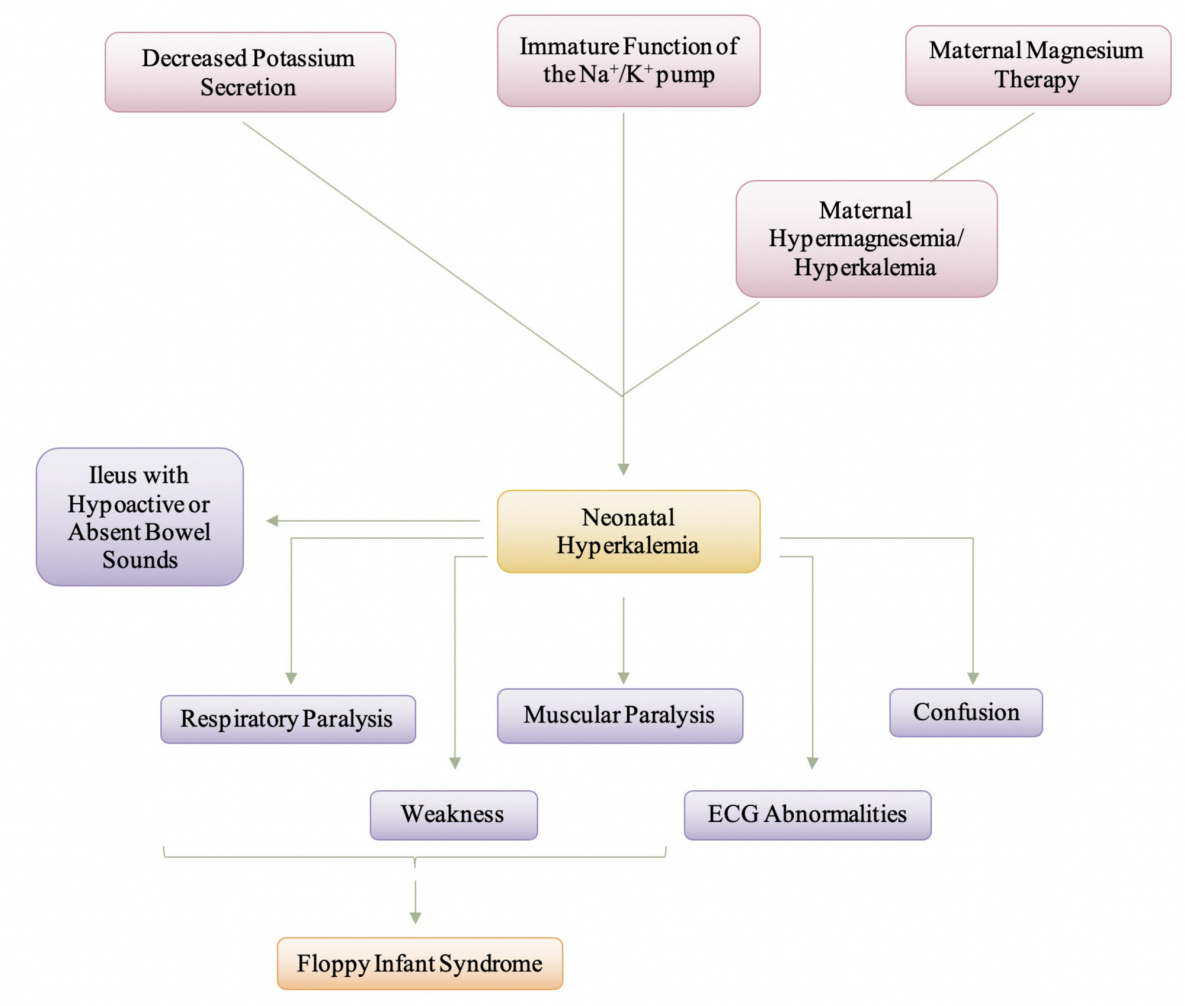
Manifestation and Pathogenesis of Neonatal Hyperkalemia

Figure 7 presents the manifestation of neonatal hyperkalemia. It is important to remember that despite what the cause of the neonatal hyperkalemia, the symptoms and manifestation will be relatively similar as the end issue becomes the increase in serum potassium that causes these symptoms to occur. The electrocardiogram (ECG) abnormalities seen with an increase in the potassium level include peaked T waves followed by a decrease in R wave amplitude, widened QRS complex, and a prolonged PR interval [44]. These ECG abnormalities lead to life-threatening cardiac arrhythmias, and that is the main reason the emergent diagnosis of hyperkalemia in infants is very important. As mentioned previously, the muscular paralysis and weakness stem from the prolonged repolarization phase during the action potential that leads to a delay in the next action potential occurring. The decreased magnitude of the action potentials due to the prolongation of the repolarization phases causes muscle weakness that can ultimately result in paralysis. That being said, this type of floppy infant syndrome presentation is reversible as treatment of hyperkalemia will decrease the serum potassium levels; thus, reversing the symptoms. The treatment of hyperkalemia causes the prolonged repolarization phase to become normal again, thus, decreasing the refractory period. The goals of hyperkalemia treatment are to antagonize the cardiac effects of potassium, reverse symptoms, and return the serum potassium level to normal while avoiding overcorrection [45]. Firstly, calcium is administered to counteract the effects of the excess on the heart [44]. Second, medications can be used to shift potassium from extracellular to intracellular compartments, and lastly, exchange resins, diuretics, or dialysis are used to remove potassium from the body [40,44]. Treating the hyperkalemia will eliminate the floppy infant syndrome, thus, making floppy baby syndrome reversible.

## Conclusions

Through this review, we attempted to not only provide insight into the complex nature of floppy infant syndrome through investigation of neuromuscular junction disorders that cause peripheral hypotonia but also offered an understanding of the presence of any treatment options possible. While the focus of this paper was on the neuromuscular junction disorders that cause floppy infant syndrome, it is imperative to understand that the exact number of incidences of floppy infant syndrome becomes challenging to calculate due to the remaining vast majority of causes of both peripheral and central hypotonia. Most of the time, instead of finding the appropriate treatment for the underlying cause of hypotonia, treatment becomes more supportive and therapeutic. For some of the causes of floppy infant syndrome, there is no actual treatment; for example, for the toxic magnesium in hypermagnesemia, other than providing fluids for hydration and initiating increased urinary output. The level of hypotonia present amongst these varying neuromuscular junction disorders is mainly dependent on the severity of the underlying abnormality; therefore, the hypotonia presents on more of a spectrum scale instead of being similar amongst the disorders. That said, the overlying cause of the neuromuscular junction disorders is around the presence of a neurosynaptic block that is either hindering the release of acetylcholine, degradation of acetylcholine by the acetylcholinesterase enzyme, or due to the lack of acetylcholine receptors present on the postsynaptic membrane. Irrespective of where in the neuromuscular junction the underlying issue occurs, the presentation remains similar, that is, it causes floppy infant syndrome. The clinical diagnosis of floppy infant syndrome encompasses the clinician observing for either the presence or absence of specific signs of hypotonia in infants. The underlying importance is for the physicians to acknowledge the hypotonia early in life and search for the underlying causative syndrome to be able to provide the appropriate intervention. Early diagnosis is imperative to ensure that those caring for the infant are made aware of any contraindications that could ultimately worsen the condition and, thus, cause irreversible consequences.
